# A Giant Pedunculated Urothelial Polyp Mimicking Bladder Mass in a Child: A Rare Case

**DOI:** 10.1155/2014/935850

**Published:** 2014-02-23

**Authors:** Mehmet Kaba, Sultan Kaba, Tacettin Yekta Kaya, Hüseyin Eren, Necip Pirinççi

**Affiliations:** ^1^Department of Urology, Faculty of Medicine, Yuzuncu Yıl University, 65000 Van, Turkey; ^2^Department of Pediatric, Faculty of Medicine, Yuzuncu Yıl University, 65100 Van, Turkey

## Abstract

Ureteral fibroepithelial polyps are rarely seen benign tumors with mesodermal origin. These polyps can involve kidney, pelvis, ureter, bladder, and urethra. The most common symptoms are hematuria and flank pain. The choice of treatment is either endoscopic or surgical resection of polyp by sparing kidney. Here, we presented a pediatric case with giant, fibroepithelial polyp that mimics bladder tumor, originating from middle segment of the ureter.

## 1. Introduction 

Ureteral fibroepithelial polyp (UFP) is a rarely seen benign tumor of mesodermal origin in infants and children [1]. Fibroepithelial polyps are associated with symptoms related to obstruction of urinary tract. The most common symptoms are hematuria and flank pain [2]. Most UFPs are observed in ureter, while 15% of UFPs are seen in renal pelvis and, less commonly, at urethra and bladder [3]. The management is simple or segmental resection with end-to-end anastomosis. Ureteroscopic excision is a less invasive and widely used alternative when compared to open surgery [2, 3]. Incomplete resection of polyp may result in tumor recurrence after surgery.

Here, we present a 14-year-old boy who presented with hematuria and had a giant fibroepithelial polyp with ureteral origin that mimics bladder tumor. To the best of our knowledge, no pediatric case with a ureteral fibroepithelial polyp in such extent that mimics bladder mass has been reported so far.

## 2. Case Report

**Figure 1:**
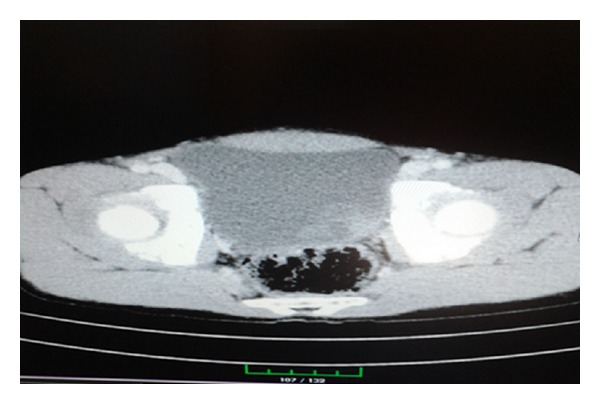
On contrasted CT, a suspicious lesion with thin septa was observed at posterolateral wall of bladder (3.5 × 3 cm in size).

A 14-year-old boy was admitted to hospital with hematuria. Results of complete blood count and biochemical test were within normal range. There was hematuria in urinalysis, but urine culture evaluation was sterile. There was no abnormal finding in his history. On abdominal sonography, a lobulated, hypoechoic mass (40 × 28 mm in size) at posterolateral wall of bladder extending to lumen was observed. On Doppler sonography, vascularization was observed at the area of mass. On CT scan, a suspicious lesion (3.5 × 3 cm in size) was observed at left posterolateral wall of bladder (Figure 1). No enlargement in lymph nodes or finding favoring metastasis was observed in the pelvic region and abdomen.

**Figure 2:**
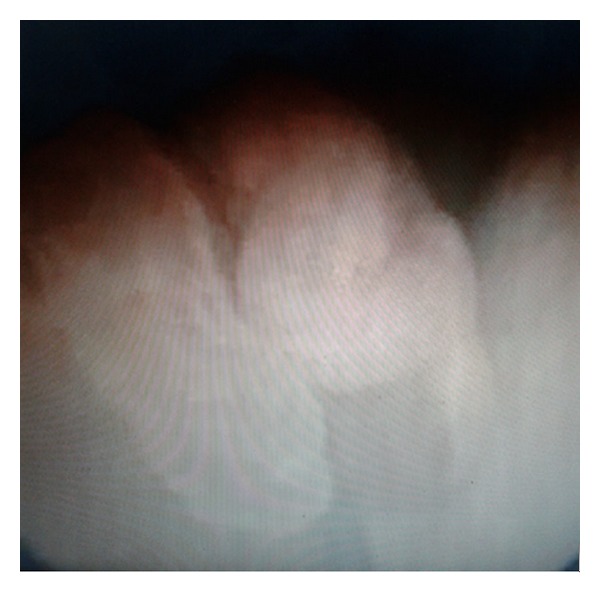
On cystoscopy, a vegetative mass (5 × 6 cm in size) at left orifice of bladder that protrudes to bladder through a stalk.

**Figure 3:**
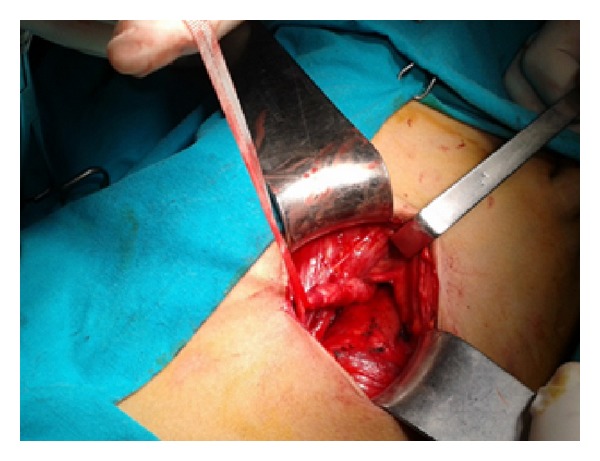
The origin of stalk at the level of iliac bifurcation.

**Figure 4:**
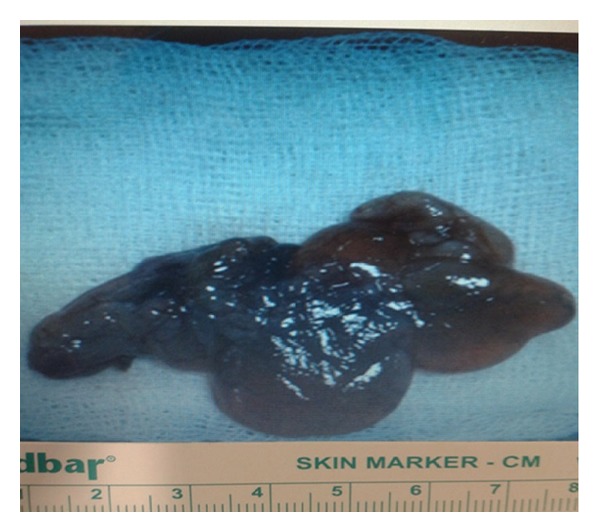
Specimen: reddish-beige tissue (8 × 4 × 1 cm in size) containing papillary projections on surface as biggest being 2.5 × 2 cm in size.

On cystoscopy, a vegetative mass (approximately 5 × 6 cm) that protruded into bladder through a stalk (Figure 2) was observed at left orifice of bladder. In the same session, ureterorenoscopic assessment was performed which revealed that the stalk extended to middle segment of ureter. However, it was failed to observe where the stalk arises at ureter. Open surgery was performed via left Gibson incision in the same session. It was seen that the stalk of polyp originated from the level of iliac bifurcation (Figure 3). After exposure of dilated region of middle ureter, ureter was opened at superior to polyp and part of ureter harboring the stalk of polyp was excised in segmental manner by preserving the distal ureter as possible (Figure 3). As frozen sections were reported as fibroepithelial polyp, the mass and its stalk were removed through bladder (Figure 3). Then, ureteral end-to-end anastomosis was performed. The part of ureter harboring the stalk of polyp was approximately 10 mm in size. Specimen was a reddish-beige tissue (8 × 4 × 1 cm in size) containing papillary projections on surface (as biggest being 2.5 × 2 cm in size) (Figure 4). Histopathological diagnosis was reported as fibroepithelial polyp. The patient was discharged on day 7 without complication. After 4 weeks, double J stent was removed. No recurrence was observed at 6-months follow-up.

## 3. Discussion

Ureteral tumors are rarely seen and they are generally malignant. However, fibroadenomatous polyps of ureter are benign tumors. They are more common in men and usually originate from left ureter [4]. It may have different clinical presentations based on localization in the ureter [4]. In rare instances, urothelial fibroadenomatous polyps may extend into the bladder cavity as far as causing misunderstanding of the surgeon [5]. Endoscopic approach is an acceptable modality of treatment with minimal complication rate and satisfactory outcomes for large fibroepithelial polyps [6–8]. Open surgeries are performed for the management of ureteral polyps extending to bladder, thus, mimicking bladder tumors [5, 9]. In our case, open surgery was preferred as the mass was rather large and the stalk of polyp could not be visualized via ureterorenoscopy.

Etiology of benign ureteral polys is unclear. It has been though that it may be either acquired due to factors such as infection, chronic irritation, obstruction, and trauma or congenital due to developmental anomaly [10–12]. In a study by Mayo Clinic, it was reported that only 27 patients with UFPs were identified between 1945 and 2008 [13]. In that series, mean age at diagnosis was 40 years. Polyps were more commonly observed at left (68%). Of the cases, 59% were proximal, while 18% were in middle segment and 18% were at distal part. There were multiple polyps in 6 cases (27%). Clinical differentiation between UFPs and malign ureteral tumors is difficult, although UFPs have a characteristic appearance. Thus, pathological evaluation is essential [14].

Complete resection is one of the most optimal methods to avoid recurrence in FEPs. The resection plus end-to-end anastomosis is surgical method of choice. In ureteral anastomosis, open procedures include ureteroneocystostomy for distal ureter, Anderson-Hynes pyeloplasty for ureteropelvic junction, and end-to-end ureteral anastomosis with or without renal mobilization. Currently, laser coagulation of origin of polyp via ureteroscopy is the most widely used method to determine histopathological diagnosis of polyps [2, 3]. Endoscopic treatment can fail to achieve complete excision, although it is associated with low rates of surgical morbidity and pain incidence and avoidance from unnecessary nephroureterectomy. Incomplete resection may result in recurrence [5]. Laparoscopic surgery is a minimal invasive technique for complete resection of polyps localized at ureter or ureteropelvic junction in pediatric patients [15]. Ureteral anastomosis may be challenging after complete laparoscopic resection of ureter in large polyps and in those with long stalk. We aimed to preserve ureter length as possible; thus, we performed minimal resection of ureter segment with whole stalk of polyp at middle segment and end-to-end ureteral anastomosis.

We excised a giant, pedunculated fibroepithelial polyp that mimics bladder tumor via open surgery. Pedunculated urothelial polyps originating from ureter should be kept in mind in the differential diagnosis of bladder mass on imaging modalities in pediatric population.
